# Classification of Movement and Inhibition Using a Hybrid BCI

**DOI:** 10.3389/fnbot.2017.00038

**Published:** 2017-08-15

**Authors:** Jennifer Chmura, Joshua Rosing, Steven Collazos, Shikha J. Goodwin

**Affiliations:** ^1^Department of Biomedical Engineering, University of Minnesota Minneapolis, MN, United States; ^2^Department of Neuroscience, University of Minnesota Minneapolis, MN, United States; ^3^Department of Integrative Biology and Physiology, University of Minnesota Minneapolis, MN, United States; ^4^School of Mathematics, University of Minnesota Minneapolis, MN, United States; ^5^Department of Neurology, University of Minnesota Medical School Minneapolis, MN, United States; ^6^Brain Sciences Center, VA Medical Center Minneapolis, MN, United States

**Keywords:** inhibition, brain computer interface, motor imagery (MI), event-related potentials (ERPs), machine learning

## Abstract

Brain-computer interfaces (BCIs) are an emerging technology that are capable of turning brain electrical activity into commands for an external device. Motor imagery (MI)—when a person imagines a motion without executing it—is widely employed in BCI devices for motor control because of the endogenous origin of its neural control mechanisms, and the similarity in brain activation to actual movements. Challenges with translating a MI-BCI into a practical device used outside laboratories include the extensive training required, often due to poor user engagement and visual feedback response delays; poor user flexibility/freedom to time the execution/inhibition of their movements, and to control the movement type (right arm vs. left leg) and characteristics (reaching vs. grabbing); and high false positive rates of motion control. Solutions to improve sensorimotor activation and user performance of MI-BCIs have been explored. Virtual reality (VR) motor-execution tasks have replaced simpler visual feedback (smiling faces, arrows) and have solved this problem to an extent. Hybrid BCIs (hBCIs) implementing an additional control signal to MI have improved user control capabilities to a limited extent. These hBCIs either fail to allow the patients to gain asynchronous control of their movements, or have a high false positive rate. We propose an immersive VR environment which provides visual feedback that is both engaging and immediate, but also uniquely engages a different cognitive process in the patient that generates event-related potentials (ERPs). These ERPs provide a key executive function for the users to execute/inhibit movements. Additionally, we propose signal processing strategies and machine learning algorithms to move BCIs toward developing long-term signal stability in patients with distinctive brain signals and capabilities to control motor signals. The hBCI itself and the VR environment we propose would help to move BCI technology outside laboratory environments for motor rehabilitation in hospitals, and potentially for controlling a prosthetic.

## Introduction

Jacques Vidal first proposed the brain-computer interface (BCI) in 1973 when he suggested translating electrical brain signals captured through electroencephalography (EEG) into computer control signals (Vidal, 1973). EEG electrodes are commonly placed using the international 10–20 placement system, called thus because each electrode is about 10–20% of the head away from its neighbor electrodes. Each pair of electrodes is then passed through an amplifier, which is typically an analog to digital amplifier now that most EEGs are read by computers (Teplan, 2002). The EEG detects biologically relevant signals that can be classified as evoked or spontaneous depending on the volitional capability of the user to control them. For instance, the performance or imagination of movements evokes changes in the brain activity that are induced by the user, while the perception of stimuli provoke spontaneous changes in the brain potentials. These breakthroughs, which have been due to successfully monitoring brain activity and translating a user's intentions into commands for a device, have led to BCIs that enable people to control a wheelchair (Castro-Borrero et al., 2012; Seáñez-González and Mussa-Ivaldi, 2014) or spell out words using their thoughts (Vourvopoulos and Bermúdez i Badia, 2016).

BCIs can also be used to control prosthetics and orthotics. One such prosthetic was developed for an amputee that correctly classified hand grasping and opening from the resting state with 83% accuracy using motor imagery (MI), in which a person imagines a motion without physically moving (Mahmoudi and Erfanian, 2002). Similarly, a BCI-controlled tetraplegic orthotic was made using visually-evoked potentials, similar to event-related potentials (ERPs), with a positive predictive value of 78% for a grasping task (Ortner et al., 2011). ERPs are neural rhythms seen in response to a stimulus, whether that is auditory, visual, or some other sense. BCI failure has been attributed to several main aspects. Some say BCIs do not have a sufficient control method for measuring brain signals, or that the hardware for measuring brain signals is insufficient (Pinegger et al., 2016), while others argue that BCIs are not reliable or robust because of inter-user variability (Jeunet et al., 2016). The accuracy of BCI-controlled prosthetics/orthotics needs to improve before patients can reliably control a BCI with a sense of agency. Machine learning can be used to better reflect the desired movement of the user, while BCI training sessions can be made more palatable by creating training games in a VR environment.

In this paper, we address this issue by proposing a hybrid BCI (hBCI) that incorporates MI signals for types of movement and ERP signals for movement inhibition. Inhibition is the ability to suppress, withhold, delay, or interrupt an action that was caused by a stimulus (Cespón et al., 2015). The manuscript is organized as follows: in Section Motor Imagery we give an overview of MI and brain signals associated with such commands; in Section ERP, we provide background information for ERPs, and how they have been used in BCI technology; in Section Hybrid BCI: Merging ERP and MI Signals, we explain the VR environments and inhibition tasks for training use of the prosthetic, as well as the machine learning mechanism for improving signal processing and classification; and in Section Conclusion, we summarize our paper and suggest future directions (cf. Box 1).

Box 1 **Future directions.**

• *Information processing in hybrid BCIs*. The concept of hybrid BCIs have been proposed in the past (Pfurtscheller, 2010; Leeb et al., 2011; Müller-Putz, 2011). One of the ideas explored in our paper is incorporating inhibitory ERPs in order to operate a BCI. Hybrid BCIs can process the recorded brain signals sequentially or simultaneously (Pfurtscheller, 2010), and the BCI in our discussion is sequential because the computer must continually switch between monitoring for MI-based signals and ERP-based signals. On the other hand, hybrid BCIs have been developed that combine SSVEP and event-related desynchronization (ERD) simultaneously for improved accuracy (Pfurtscheller, 2010). If ERPs are not suitable for our discussed BCI, inhibitory-related ERD (or ERS) might be more appropriate. Further work on the implementation and seeing the advantages, if any, in a simultaneous BCI would be necessary.

• *Selecting among responses to be inhibited*. In this manuscript, by the very nature of the EEG signals used and the tasks employed in the VR environment, all activity must be stopped, so inhibition is non-selective in this case. In realistic situations, we often have to choose among several possible responses which ones to inhibit. Some investigators have suggested further research into this issue of selectivity (Verbruggen and Logan, 2008), and others have suggested tasks for such a paradigm (Ko and Miller, 2011). Further research is needed into components of EEG-signals associated with these tasks, as well implementation of them in a VR environment. This research might lead to improved hybrid BCIs that can discriminate among responses, whether environmentally induced or self-generated, the user wishes to inhibit.

• *Performance measures for the games*. There are metrics that are employed in different prosthetic modalities in order to determine the efficacy of the prosthetic for the user. These metrics may have an impact on whether insurance can cover the cost of the prosthetic, as in the case of K-levels for Medicare. As the VR environment is part of the rehabilitation of the patient, it would be desirable to determine the response of the user to the VR environment and tangible ways for how the inhibition tasks are aiding the patient in recovery as well as during recalibration of the algorithms.

## Motor imagery

Within various frequency bands, there are amplitude changes in cortical rhythms that are associated with motor movements and imagination. Before moving, there is a specific blocking or desynchronization of 8–13 Hz (mu) and 14–25 Hz (beta) rhythms, called event-related desynchronization (ERD) (Nam et al., 2011). Termination of the movement shows event-related synchronization (ERS) within the 15–25 Hz beta bands in the precentral region of the brain (Nam et al., 2011). The post-movement beta ERS has been debated heavily. While some have found that ERS is dominant over the contralateral precentral cortex, others have also found it occurring on the ipsilateral side (Nam et al., 2011). Hence, decoding a “stop” signal from MI is difficult because the ERS signal varies not only spatiotemporally between individuals, but also between movements. Furthermore, discriminating between more than two states can be difficult when using ERD patterns, because many complex memory processes and tasks can cause desynchronization in alpha band rhythms (Pfurtscheller, 2010; Müller-Putz, 2011; Ortner et al., 2011).

## ERP

Event-related potentials (ERPs) arise in response to stimuli. There are at least two known ERPs that are related to inhibition and veto—N200 (N2) and P300 (P3) (Greenhouse and Wessel, 2013)—which have been associated with reactive inhibitory control processes when performing go/no-go (GNG) and stop signal tasks (SST). Research in the literature suggests N2 arises from the extrastriate temporo-occipital and associated parietal cortical regions (Hong et al., 2009). Furthermore, N2 is displayed by fronto-central negativity 200–300 ms after the stimulus is presented, while P3 is a positive response in the fronto-central to centro-parietal area following N2 by about 150 ms (Huster et al., 2013). Both N2 and P3 are enhanced during motor inhibition, and while it is not clear what differentiates them, there is evidence that N2 is associated with control over a response plan, while P3 reflects evaluation of motor response inhibition (Greenhouse and Wessel, 2013). A study by Wessel et al. looked at when P3 occurs relative to the ability to inhibit an action, and found that the P3 onset and latency appear to be related to whether or not the action can be vetoed. When P3 occurred before a certain time (about 200 ms following the stop signal) a successful stop occurred, whereas if it was later a failed stop occurred. From this, there are three hypotheses for how P3 is connected to successful stop-reaction time (SSRT): the onset latency is positively correlated with SSRT; the onset time point is better correlated with SSRT than the peak time for P3; and P3 onset is earlier in successful trials compared to failed trials (Wessel and Aron, 2015).

The go/no-go (GNG) and stop signal task (SST) are robust and reliable measures for inhibition, and are the preferred methods for analyzing N2 and P3 ERPs in motor inhibition for healthy individuals. The resting-state prior to the response inhibition signal has been used to predict the success of motor inhibition in healthy individuals with a 95% prediction accuracy using one classifier (Chikara and Ko, 2016). A challenge of using these signals for BCI-controlled prosthetics is that they require attentiveness to the stop signal and engagement of response inhibition, which is also reliant on external cues from a controlled game, as opposed to the user's self-pacing which better reflects volition. A GNG task involves showing two equiprobable stimuli, and having the subject either respond or withhold the response, while SSTs involve the subject responding to a shown stimulus, unless an imperative stimulus is shown that prompts them to withhold their response (Thomas et al., 2008). The robustness and reliability of GNG and SST, and their wide use in research in healthy individuals could make them useful training tasks for a hybrid BCI.

## Hybrid BCI: merging ERP and MI signals

The hybrid BCI (hBCI) was introduced as a device which combines multiple existing inputs, including MI and ERPs, by either switching them on and off or fusing them together. The hBCI has many components to it: user driven input signals, control systems from the environment, and feature extraction and classification methods (Pfurtscheller, 2010; Müller-Putz, 2011). Additionally, some research groups have developed new paradigms suitable for hBCIs that result in higher classification accuracies of certain ERPs (Wang et al., 2015). The idea of a hybrid BCI in orthotic control has been previously proposed, where the user controls movement through volitionally generated SSVEP signals from a screen with a grid of flickering light frequencies (Ortner et al., 2011). A “brain switch” using another mental process, like ERPs, would be used to turn on/off the SSVEP grid. However, we instead propose using MI signals to distinguish between movements, and using ERP signals to determine user intention to start or stop a movement. This hybrid BCI design uses the signals in our natural decision making and motor movement intuitively. This could also improve training for hybrid BCI use, as well as improve movement inhibition encoding.

ERPs and MI can be combined in BCIs for movement using training for both the patient and the machine learning algorithm. The user initially sets the baseline signal processing characteristics and classifiers for the BCI by going through GNG and SST tasks in a VR environment that are disguised as games. These baseline characteristics are used to provide visual and metric feedback while collecting data on the patient's MI and ERPs. Once a sufficient amount of data is collected, a machine learning algorithm is used to optimize signal processing and classification. The classifier distinguishes between movements like reaching or grasping using MI, as well as determining whether the signal is inhibited or not using ERPs. By continuing to train, the user improves the ability of their BCI to successfully interpret their movement intentions and inhibition of movement. Continuing to use the algorithm to update the classifier will help the BCI to retain its accuracy over time, even with changes in the user's brain patterns.

## Virtual reality environment

GNG and SST VR training would involve immersing the user into gamified tasks that require moving only when allowed, and having to stop when presented with a cue. The gamification of tasks has been suggested as a way of making them more engaging to users. Similarly, task complexity has also been used to improve user performance in both MI and ERP BCIs (Jin et al., 2017; Qiu et al., 2017). This suggests that a gamified task of sufficient complexity would aid in user training of a BCI task. In addition, video games themselves have been shown to promote learning of perceptual tasks, which further increases the usefulness and suitability of virtual games to BCI training for prosthetic use or motor control (Feng et al., 2007; Deveau et al., 2014). To increase immersion in the VR environment within the games, a VR headset would be used. The games would also be designed for a first-person, 3D environment to heighten the sense of agency. It has previously been shown that combining training with VR and video games improves BCI performance (Lotte et al., 2013). An example of this would be a game in which the user attempts to steal cookies from a jar under the protection of a watchful parent. The user would be allowed to move their hand toward the cookie jar only when the parent is looking away. The “go” cue is then when the parent turns to look away, while the “stop” cue is when the parent turns to look at the player. By varying the timing of the cues, GNG and SST tasks can be performed. The motions that could be performed by the patient using MI include reaching with the left or right arm, or grasping onto a cookie. The player could gain points for successfully stealing a cookie, but could lose points if they fail to stop moving and are caught by the parent. A similar scenario can be set up for the reaching and grasping motion of different objects, like a cup or a pencil, to adapt to the needs of the patient. Adapting the application of the BCI to the user's needs is especially important for upper-limb amputees, who vary in the actions they require for daily life, as well as in their motor and cognitive abilities. Hence, the VR environment can be modified to encode different movements based on the user desired functionality of the prosthetic. Instead of showing the same, repetitive training protocol, the user will be rewarded with points for their success, and engage in the game while also training the BCI on their MI and ERP signals. Time taken to complete a task would vary, with a shorter, more simple task like a reaching and grabbing task taking as little as 30 s, while a longer task, such as one made up of repeated movements or with a wait time, would take longer to complete.

## Signal processing, classification, and machine learning

**Figure 1:**
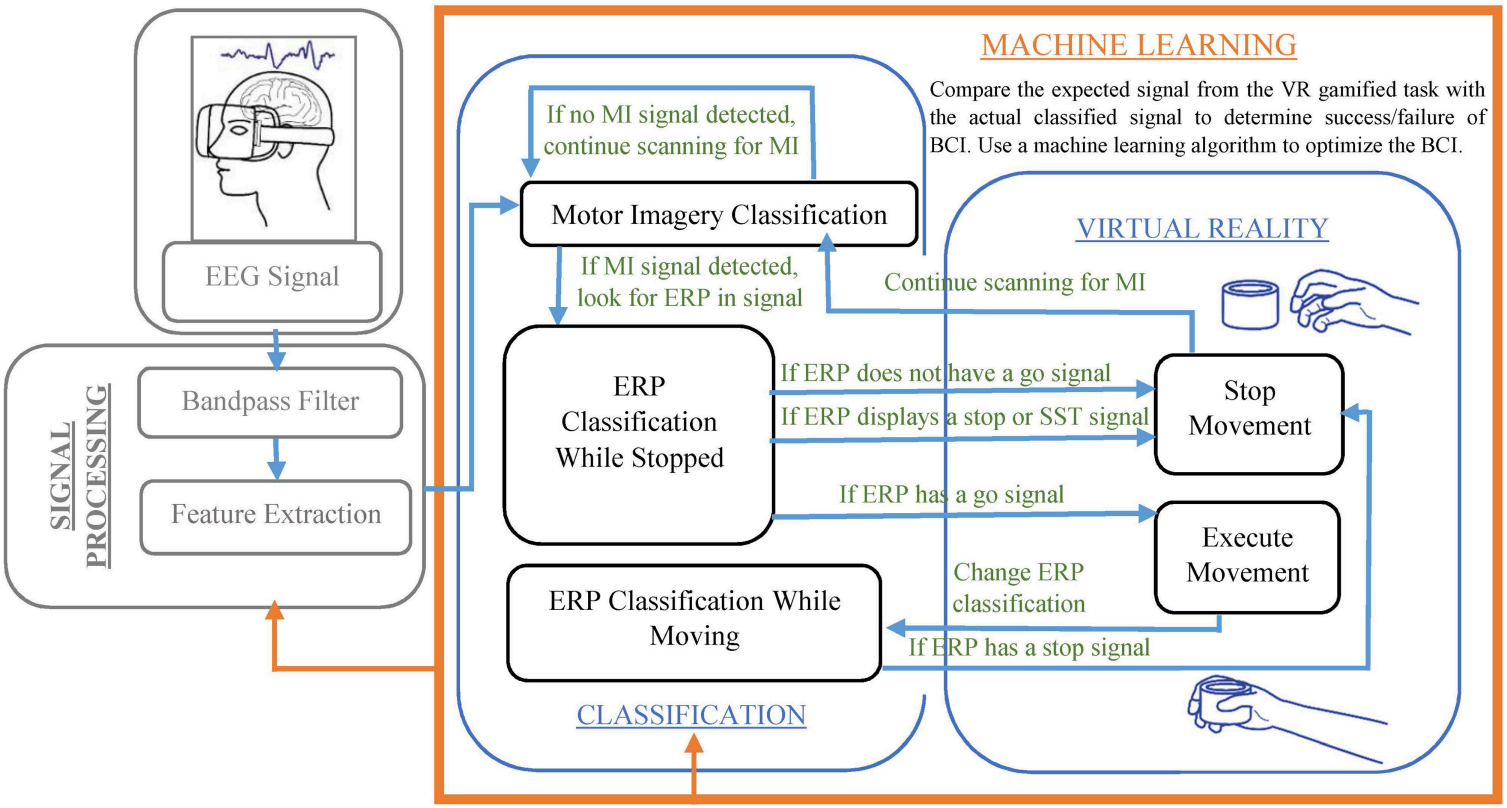
VR training of a hybrid BCI using MI and ERP inputs and a machine learning algorithm.

**Figure 2:**
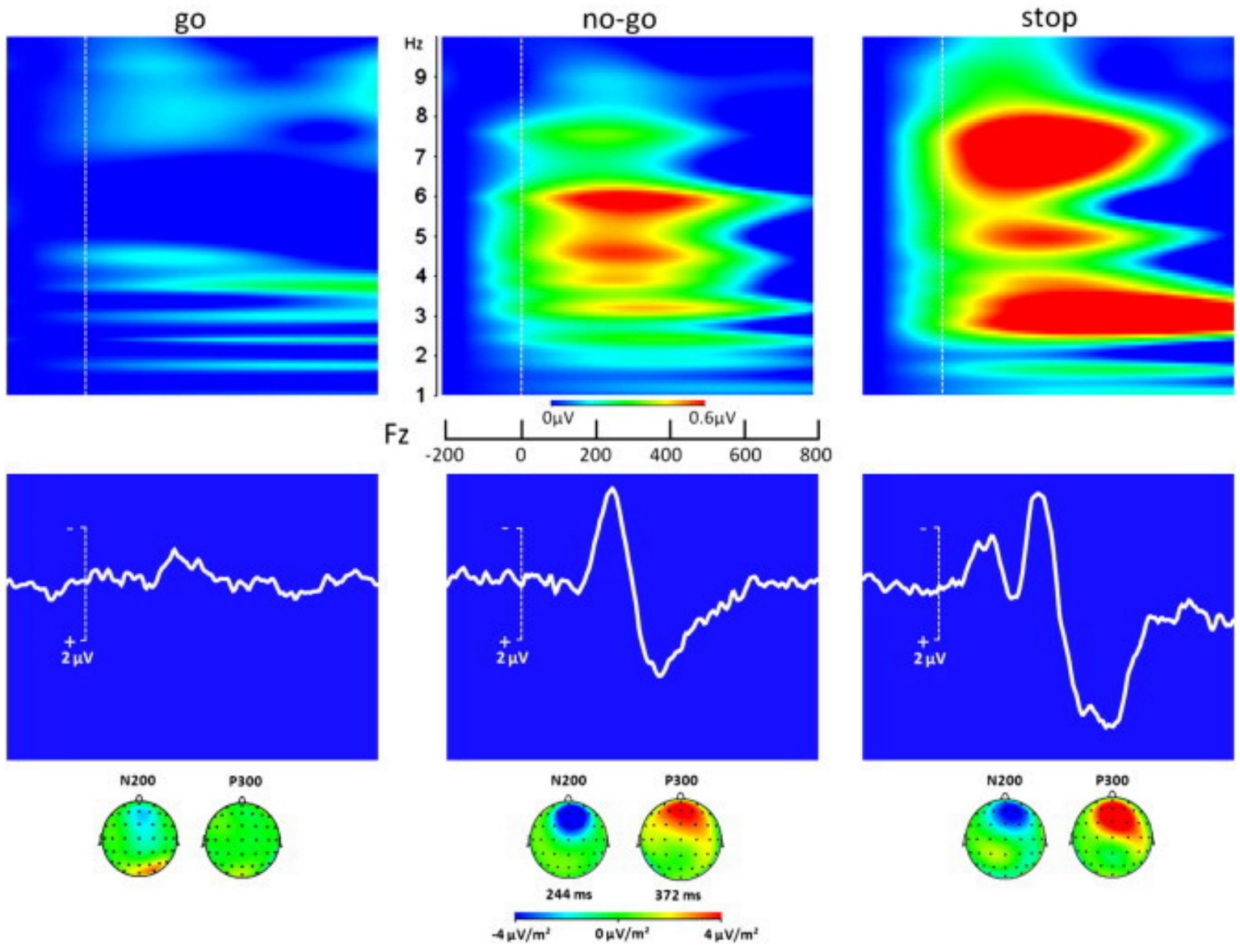
ERP responses in a subject performing go, no-go, and stop tasks. The middle row shows the ERPs; the top row shows the associated time-frequency decompositions; and the bottom row shows how the N200 and P300 potentials are distributed over the scalp (Adapted with permission from Huster et al., 2013).

The BCI would perform real-time computation of a continuous control signal for features created by MI. The quantification measurements of MI include the ERD and ERS, band power, inter-trial variance, temporal spectral evolution, autoregressive models and spectral decomposition, task-related power changes, and others (Gao et al., 2016). In a hybrid BCI, the extracted features would then be classified into what movement they indicate (using MI), and if a movement is indicated it then proceeds to see if the movement is initiated or inhibited (using ERP). The signal would be actively analyzed for MI movements, including left and right arm reaching, throughout the VR training. For instance, when imagining a movement of the left hand, a characteristic decrease in EEG beta-rhythm power is seen over the right motor cortex (Pfurtscheller, 2010; Nam et al., 2011; Tombini et al., 2012). Then, the EEG signal would begin to be analyzed for ERPs that indicate motion inhibition. If a signal is generated similar to what is seen in the SST training, the motion is never begun, while if it is similar to what is seen in the GNG training or the go trials of the SST training, the motion is started. If a stop signal is seen as would be expected from GNG stop signals, the motion is stopped. While MI classifies the type of motion done, ERP classifies when the motion is started or stopped. The details of using classification for determining visual feedback in the VR is shown in Figure 1. A typical ERP signal is shown in Figure 2 (taken from Huster et al., 2013).

There are two steps of signal processing that would be modified by machine learning. The first step is the bandpass filter. An adaptive filter bank was proposed by Thomas et al. to combat the subject-specific variation in alpha and beta band activity (Thomas et al., 2008). Their method involves using a power spectral density along different time windows, and a time-frequency Fisher ratio to determine which frequency components contain the most information. The optimal band window would carry all of the information necessary to correctly categorize signal. While this is computationally heavy, this limits the amount of data the software will have to process, and it is necessary due to intersubject variability in MI and ERP signals. The second step is the feature extraction. The EEG signal from each filter band would be applied with a common spatial pattern (CSP) transformation in order to extract features for MI. If MI indicates a motion, then the ERP signals are extracted, too. These features would be weighted for both the probability of their values indicating a successful motion, as well as their probability of being successful when compared against other features. Bayesian classifiers are a common classifier found in BCIs. They use the probabilities of the events to improve the weight of the feature when classifying a motion or direction (Müller-Putz, 2011). Studies often investigate classifiers with feature vectors of high dimensions. However, the cost, computational intensiveness, and processing time is not conducive with our proposed VR training game application. An ideal classifier would instead use low dimension feature vectors so that the machine learning can iterate quickly with the signal processing and feature extraction. Naive Bayes and support vector machines are examples of classifiers that are capable of handling low dimension feature vectors accurately and quickly.

The machine learning algorithm compiles the different features of MI and ERP that were extracted over an array of smaller frequency bands for each of the movements and inhibitions. A smaller frequency band may better display the change in power, just as certain features and values can better indicate success compared to others. The success of each feature within a frequency band will be consolidated and used to both weight the feature against other features, and for values within the feature itself. For instance, if the CSP of the ERP related to stopping failed to indicate stopping using the classifier, the CSP is weighted less in comparison to more successful features, like the slope, in a future training session. If a value of CSP occurs more frequently than other values, that value is weighted more heavily for success, as well. These weights can be determined using Bayesian probability. Both of these feature weights improve the classification of the movement or inhibition. The context of the VR task indicates whether there is a success to a movement. For example, failing to reach for the cookie jar when prompted, or failing to stop when the parent looks at the player, would indicate either that the patient themselves failed to create the motion or recognize the stop signal, or that the BCI did not properly recognize the signals they generated. With machine learning, the BCI optimizes both the frequency band and the weight of the features extracted for signal processing, thus customizing the algorithm to the user.

## Conclusion

BCIs for the control of motion in prosthetics, orthotics, and virtual environments are a promising technology for restoring motor function including in upper-limb amputees and stroke patients. To improve classification of movements as well as inhibition, we presented a hybrid BCI that uses both MI and ERP within a virtual reality environment training. The virtual reality environment uses gamified GNG and SST tasks to improve the training of the user to their BCI, while the machine learning aspect improves the accuracy of the BCI in decoding the user's intention. The future directions of this technology are shown in Box 1.

## Author contributions

All authors listed have made a substantial, direct and intellectual contribution to the work, and approved it for publication.

### Conflict of interest statement

The authors declare that the research was conducted in the absence of any commercial or financial relationships that could be construed as a potential conflict of interest.
